# GDF‐15 Levels in Cirrhosis Are Linked to Hepatic Fibrogenesis, Bacterial Translocation, and Worse Clinical Outcomes

**DOI:** 10.1111/liv.70516

**Published:** 2026-01-19

**Authors:** Benedikt Silvester Hofer, Thomas Perkmann, Ksenia Brusilovskaya, Lorenz Balcar, Marlene Hintersteininger, Georg Kramer, Benedikt Simbrunner, Esther Caparros, Rubén Francés, Beate Eichelberger, Silvia Lee, Kerstin Zinober, Benjamin Bödendorfer, Borka Radovanovic‐Petrova, Paul Thöne, Christian Sebesta, Mathias Jachs, Lukas Hartl, Philipp Schwabl, Mattias Mandorfer, Simon Panzer, Thomas Reiberger, Thomas Gremmel

**Affiliations:** ^1^ Division of Gastroenterology and Hepatology, Department of Medicine III Medical University of Vienna Vienna Austria; ^2^ Division of Gastroenterology and Hepatology, Department of Medicine III, Vienna Hepatic Hemodynamic Laboratory Medical University of Vienna Vienna Austria; ^3^ Christian Doppler Lab for Portal Hypertension and Liver Fibrosis Medical University of Vienna Vienna Austria; ^4^ Clinical Research Group (CRG) MOTION Medical University of Vienna Vienna Austria; ^5^ Department of Laboratory Medicine Medical University of Vienna Vienna Austria; ^6^ Center for Molecular Medicine (CeMM) of the Austrian Academy of Sciences Vienna Austria; ^7^ CIBEREHD, Instituto de Salud Carlos III Madrid Spain; ^8^ Department of Clinical Medicine Hepatic and Intestinal Immunobiology Group, Miguel Hernández University Elche Spain; ^9^ Instituto IDIBE, Miguel Hernández University Elche Spain; ^10^ Instituto ISABIAL, Hospital General Universitario de Alicante Alicante Spain; ^11^ Department of Blood Group Serology and Transfusion Medicine Medical University of Vienna Vienna Austria; ^12^ Department of Internal Medicine II Medical University of Vienna Vienna Austria; ^13^ Institute of Cardiovascular Pharmacotherapy and Interventional Cardiology, Karl Landsteiner Society St. Pölten Austria; ^14^ Department of Internal Medicine I Cardiology and Intensive Care Medicine, Landesklinikum Mistelbach‐Gänserndorf Mistelbach Austria; ^15^ Karl Landsteiner University of Health Sciences Krems Austria

**Keywords:** advanced chronic liver disease, blood platelets, haemostasis, hepatic decompensation, portal hypertension

## Abstract

**Background &Aims:**

Growth differentiation factor‐15 (GDF‐15), a cell stress‐induced cytokine, is implicated in liver disease pathophysiology. We investigated GDF‐15 in cirrhosis, focusing on its association with disease‐driving pathomechanisms, platelet function, hepatic decompensation, and mortality.

**Methods:**

We included patients with cirrhosis undergoing hepatic venous pressure gradient (HVPG) measurement at the Vienna General Hospital. Platelet surface P‐selectin and glycoprotein IIb/IIIa (GPIIb/IIIa) expression after agonist stimulation were assessed by flow cytometry as platelet activation markers. GDF‐15 serum levels were quantified by electrochemiluminescence immunoassay.

**Results:**

Among 106 patients (median age 55.1 years; 70.8% male), median GDF‐15 was 2880 (1850–4770) pg/mL. GDF‐15 correlated with hepatic dysfunction (MELD Spearman's *ρ*: 0.50; albumin *ρ*: −0.57), HVPG (*ρ*: 0.47), systemic inflammation (C‐reactive protein *ρ*: 0.45; interleukin 6 [IL‐6] *ρ*: 0.55; procalcitonin *ρ*: 0.58), liver stiffness (*ρ*: 0.67) and enhanced liver fibrosis test (*ρ*: 0.64) (all *p* < 0.001). GDF‐15 was higher in patients with detectable bacterial DNA in blood (3520 vs. 2250 pg/mL; *p* < 0.001) and correlated with lipopolysaccharide (*ρ*: 0.34; *p* = 0.010) and lipoteichoic acid (*ρ*: 0.37; *p* = 0.004). Platelet activation was not linked to GDF‐15 after adjusting for liver disease severity, yet patients with undetectable GPIIb/IIIa activation after stimulation showed significantly higher GDF‐15. Over a median follow‐up of 51.5 (26.0–58.2) months, 38 patients decompensated and 21 died (61.9% liver‐related). GDF‐15 (aHR per 100 pg/mL: 1.015; 95% CI: 1.004–1.026; *p* = 0.007) predicted decompensation risk independently of HVPG, MELD, albumin and IL‐6. Similarly, GDF‐15 was associated with higher risk of all‐cause (HR: 1.019; 95% CI: 1.009–1.029; *p* < 0.001) and liver‐related mortality (HR: 1.019; 95% CI: 1.007–1.032; *p* = 0.002).

**Conclusions:**

GDF‐15 is a promising biomarker in cirrhosis that reflects disease‐driving pathomechanisms and independently predicts decompensation and mortality.

## Lay Summary

Growth differentiation factor‐15 (GDF‐15) increases under conditions of cell stress and is elevated in various chronic diseases, including cancer and heart disease. In liver disease, the role of GDF‐15 is unclear, with prior findings showing both protective effects and associations with more pronounced liver disease severity and worse outcomes. In the current study of patients with cirrhosis, higher GDF‐15 levels primarily reflected ongoing liver scarring, inflammation, and bacterial translocation from the gut, and were linked to a higher risk of liver‐related complications and death.

**Abbreviations**
ALD: alcohol‐related liver disease
ALT: alanine transaminase
AST: aspartate transaminase
BMI: body mass index
CRP: C‐reactive protein
ELF: enhanced liver fibrosis test
GDF‐15: growth differentiation factor‐15
GPIIb/IIIa: glycoprotein IIb/IIIa
HA: hyaluronic acid
HCC: hepatocellular carcinoma
HE: hepatic encephalopathy
HR: hazard ratio
HVPG: hepatic venous pressure gradient
IL‐6: interleukin 6
INR: international normalised ratio
LBP: lipopolysaccharide‐binding protein
LPS: lipopolysaccharide
LSM: liver stiffness measurements by vibration‐controlled transient elastography
LTA: lipoteichoic acid
MASLD: metabolic dysfunction‐associated steatotic liver disease
MELD: model for end‐stage liver disease
MFI: median fluorescence intensity
PAR: protease‐activated receptor
PBS: phosphate‐buffered saline
PCT: procalcitonin
PIIINP: procollagen III amino‐terminal peptide
TGF‐β: transforming growth factor‐beta
TIMP: tissue inhibitor of metalloproteinase‐1
TIPS: transjugular intrahepatic portosystemic shunt
vWF: von Willebrand factor

## 1 Introduction

Growth differentiation factor‐15 (GDF‐15), a cytokine of the transforming growth factor‐beta (TGF‐β) superfamily, is upregulated in response to cellular stress and inflammation, where it exerts anti‐inflammatory effects and induces tissue tolerance—hence its previous name, ‘macrophage inhibitory cytokine‐1’ [1, 2, 3].

From a clinical perspective, elevated GDF‐15 has been associated with increased all‐cause mortality [4] and implicated in numerous chronic diseases, reflecting its role in orchestrating inflammation in the presence of cellular stress. For example, GDF‐15 has been shown to play a role in cancer development and progression as it was, among other things, found to predict prostate cancer [5], influence the prognosis of colorectal malignancies [6] and induce cancer‐related cachexia [7]. Similarly, GDF‐15 is a well‐established prognostic biomarker in cardiovascular medicine and predicts adverse outcomes after myocardial infarction [8] and future cardiovascular events [9]. GDF‐15 has also been found to predict bleeding complications in atrial fibrillation [10] or acute coronary syndrome [11], which may be explained by the inhibitory effects of GDF‐15 on platelet activation [12, 13, 14, 15].

Given its broad regulatory effects, increasing attention has turned to the role of GDF‐15 in liver disease, yet its mechanistic impact remains incompletely understood. On the one hand, studies have not only linked elevated GDF‐15 levels to more advanced hepatic fibrosis [16, 17] and the presence of hepatic decompensation [18], but also to hepatocyte senescence, increased liver cancer risk [19], and decreased survival in alcohol‐related hepatitis [20]. On the other hand, preclinical data suggest GDF‐15 ameliorates liver disease progression by decreasing the expression of proinflammatory cytokines [21] and suppressing genes related to fibrogenesis [22].

Considering these controversial findings and the potential involvement of GDF‐15 in orchestrating multiple underlying pathophysiological pathways in liver disease [23], further data is clearly needed. Thus, we decided to measure GDF‐15 in patients with cirrhosis to investigate (i) its association with key liver disease‐driving mechanisms and portal hypertension, (ii) its relationship with platelet function, and (iii) its prognostic value for hepatic decompensation, liver‐related death and all‐cause mortality.

## 2 Methods

### 2.1 Study Cohort and Study Design

This single‐centre study included prospectively recruited patients with cirrhosis undergoing hepatic venous pressure gradient (HVPG) measurement and same‐day platelet function testing at the Vienna General Hospital between July 2019 and December 2020. Serum GDF‐15 levels were quantified from stored biobank samples collected on the day of HVPG measurement.

Cirrhosis was diagnosed based on a combination of liver histology (where available), non‐invasive tests [24] and typical biochemical/imaging findings. Patients were excluded in case of ongoing anticoagulation/antiplatelet therapy, acute‐on‐chronic liver failure or infection, a history of transjugular intrahepatic portosystemic shunt (TIPS) insertion or liver transplantation, current/previous portal vein thrombosis, and hepatocellular carcinoma (HCC) or active non‐hepatic malignancies at the time of HVPG measurement.

All patients provided informed consent prior to study inclusion. The study was approved by the local ethics committee of the Medical University of Vienna (2317/2019) and conducted in accordance with the 1964 Declaration of Helsinki and its later amendments.

### 2.2 HVPG Measurement, Liver Stiffness Assessment and Blood Sampling

HVPG assessments were conducted in accordance with a published protocol [25] and international [24] and national [26] guidelines. Following HVPG measurement, blood was drawn from the central venous access [27]. Liver stiffness measurements (LSM) were conducted using vibration‐controlled transient elastography (FibroScan, Echosens, Paris, France) according to a standardised protocol. LSM values were only analysed if reliability criteria were met (IQR/median ≤ 30%) [28].

Serum samples for GDF‐15 measurements were collected in serum tubes (Greiner Bio‐One, Austria), centrifuged, and stored at −80°C until analysis. Samples for the assessment of platelet function were collected in 3.2% sodium citrate tubes (Greiner Bio‐One, Austria), processed within 30 min, and analyzed using flow cytometry within 60 min (same methodology and operator [KB] throughout the study). Further samples for the analysis of blood‐based biomarkers were collected according to standard operating procedures and analysed at the ISO‐certified Department of Laboratory Medicine of the Vienna General Hospital.

Bacterial translocation in blood was assessed by lipopolysaccharide‐binding protein (LBP) in all patients and by lipopolysaccharide (LPS), lipoteichoic acid (LTA), and the presence of bacterial DNA in a subgroup of patients. Further details on measurement techniques are provided in the Supporting Informations.

### 2.3 Assessment of GDF‐15 and Platelet Function

GDF‐15 concentrations in serum were measured using a cobas 8000 modular analyser (cobas e 801 module) with the CE‐marked Elecsys GDF‐15 electrochemiluminescence sandwich immunoassay (Roche Diagnostics, Rotkreuz, Switzerland). The assay followed the manufacturer's instructions. It is based on a two‐step sandwich method with biotinylated and ruthenylated monoclonal antibodies targeting GDF‐15, detected through electrochemiluminescence. The assay's analytical range is 400–20 000 pg/mL.

Platelet reactivity to specific agonists was assessed by flow cytometry as a platelet‐count independent method [29]. A detailed description of the methodology can be found in the Supporting Informations. In brief, the expression of P‐selectin and activated glycoprotein IIb/IIIa (GPIIb/IIIa) on the platelet surface was measured following stimulation with (i) SFLLRN (protease‐activated receptor (PAR)‐1 agonist), (ii) AYPGKF (PAR‐4 agonist), or (iii) epinephrine, using a FACS Canto II flow cytometer (Becton Dickinson, USA). The obtained median fluorescence intensity (MFI) values were used for further analyses.

### 2.4 Follow‐Up and Events of Interest

Patients entered the study at baseline HVPG assessment and were subsequently followed prospectively at regular intervals in the cirrhosis outpatient clinic of the Vienna General Hospital until either (i) liver transplantation, (ii) the last available visit (until 03/2025), or (iii) death, whichever occurred first. All inpatient stays during follow‐up were documented and analysed. HCC screening was conducted in accordance with international guidelines [30].

The primary outcome parameters were first/further hepatic decompensation, liver‐related death, and all‐cause mortality. Hepatic decompensation was defined according to Baveno VII criteria [24], as outlined in the Supporting Informations. Date and cause of death were acquired from the patient's electronic health records and a nationwide mortality database (Statistik Austria). Death was classified as liver‐related if it occurred as a direct consequence of the underlying liver disease or due to liver disease progression, including HCC.

### 2.5 Statistical Analysis

Statistical analyses were performed using R 4.5.0 (R Core Team, R Foundation for Statistical Computing, Vienna, Austria).

Spearman correlation coefficients were used to assess the correlation between continuous variables. Locally estimated scatterplot smoothing was used within the depiction of correlation analyses. Multivariable linear regression models were used to assess the association of multiple parameters with GDF‐15 in exploratory analyses. All models were assessed to meet assumptions of linearity, homoscedasticity, and normality of residuals by visual inspection of diagnostic plots. Multicollinearity was assessed by variance inflation factors.

Median follow‐up time was calculated by the reverse Kaplan–Meier method. The prognostic impact of GDF‐15 levels was analysed based on uni‐ and multi‐variable cause‐specific Cox regression models. The multivariable model assessing the impact of GDF‐15 on the risk of first/further decompensation included parameters known to influence decompensation risk: portal hypertension severity (i.e., HVPG), hepatic dysfunction (i.e., MELD, albumin) and systemic inflammation (i.e., IL‐6). Multivariable models assessing all‐cause and liver‐related mortality included HVPG, albumin or IL‐6 in multiple distinct models to account for the limited number of events. Age was additionally included in all models assessing (independent) risk factors for all‐cause mortality. Multicollinearity was evaluated by variance inflation factor, and the proportional hazards assumption was assessed by Schoenfeld residuals. In addition to loss of follow‐up, liver transplantation and death, which were used as censoring events in all outcome analyses, patients were censored upon TIPS insertion within the analysis of first/further decompensation. Within the analysis of major bleeding events, patients were additionally censored if long‐term anticoagulation was required following a thromboembolic event.

Outcome analyses were depicted using Kaplan–Meier curves and compared using the log‐rank test. GDF‐15 values were dichotomised based on ideal prognostic cut‐off values calculated via maximally selected rank statistics.

Two‐sided *p*‐values < 0.05 were considered statistically significant. Further details on statistical analyses are specified in the Supporting Informations.

## 3 Results

### 3.1 Characteristics of Study Cohort

A total of 106 patients undergoing same‐day HVPG, platelet function, biomarker and GDF‐15 assessment were included (Figure S1). Median age was 55.1 years, and 70.8% were male. Alcohol‐related liver disease (ALD; 49.1%) and viral hepatitis (22.6%) were the most prevalent aetiologies.

At baseline, 39.6% of patients were compensated, while 60.4% had a history of decompensation. Median MELD was 11, with 57.5% of patients classified as Child‐Pugh A, 32.1% as Child‐Pugh B, and 10.4% as Child‐Pugh C. Median HVPG was 18 mmHg, and clinically significant portal hypertension was present in 85.8%. Median GDF‐15 levels were 2880 (IQR: 1850–4770) pg/mL. Measured values ranged from 400 pg/mL to 15 560 pg/mL, all within the range of the assay. Detailed baseline characteristics are provided in Table 1.

**TABLE 1:** Baseline characteristics of the overall cohort and stratified by GDF‐15 levels.

	Full cohort (*n* = 106)	GDF‐15 low (*n* = 76)	GDF‐15 high (*n* = 30)	*p*
GDF‐15 (pg/mL)	2880 (1850–4770)	2230 (1590–2980)	6990 (5270–8880)	**< 0.001**
Age (years)	55.1 (46.4–62.7)	56.1 (47.0–62.6)	54.3 (43.5–65.0)	0.965
Sex (male)	75 (70.8%)	53 (69.7%)	22 (73.3%)	0.714
BMI (kg/m^2^)	26.0 (22.7–30.2)	26.0 (23.1–30.6)	25.8 (22.1–27.8)	0.180
Aetiology of cirrhosis				
ALD	52 (49.1%)	32 (42.1%)	20 (66.7%)	**0.019**
Viral hepatitis	24 (22.6%)	22 (28.9%)	2 (6.7%)	
Cholestatic disease	8 (7.5%)	5 (6.6%)	3 (10.0%)	
MASLD	7 (6.6%)	7 (9.2%)	0 (0.0%)	
Other	15 (14.2%)	10 (13.2%)	5 (16.7%)	
Decompensated disease	64 (60.4%)	39 (51.3%)	25 (83.3%)	**0.002**
HVPG (mmHg)	18 (12–21)	17 (11–20)	20 (17–23)	**< 0.001**
CSPH	91 (85.8%)	63 (82.9%)	28 (93.3%)	0.223
HVPG ≥ 16 mmHg	64 (60.4%)	40 (52.6%)	24 (80.0%)	**0.009**
MELD	11 (9–15)	10 (8–13)	15 (12–18)	**< 0.001**
Child‐Pugh stage (A/B/C)	57.5%/32.1%/10.4%	72.4%/19.7%/7.9%	20.0%/63.3%/16.7%	**< 0.001**
Albumin (g/L)	37.0 (33.6–40.4)	38.7 (35.5–40.7)	33.6 (29.9–36.6)	**< 0.001**
Bilirubin (mg/dL)	1.12 (0.75–1.99)	1.06 (0.74–1.59)	1.77 (0.87–3.38)	**0.015**
INR	1.4 (1.2–1.5)	1.3 (1.2–1.5)	1.4 (1.2–1.5)	0.151
Creatinine (mg/dL)	0.72 (0.61–0.91)	0.71 (0.60–0.85)	0.79 (0.62–1.02)	0.071
Sodium (mmol/L)	139 (137–140)	139 (137–140)	138 (136–139)	**0.009**
AST (U/L)	40 (29–56)	37 (28–50)	56 (31–81)	**0.012**
ALT (U/L)	30 (21–40)	30 (21–45)	30 (24–40)	0.838
CRP (mg/dL)	0.24 (0.09–0.62)	0.18 (0.08–0.47)	0.61 (0.31–1.15)	**< 0.001**
IL‐6 (pg/mL)	7.1 (3.8–13.3)	5.4 (3.1–9.9)	16.0 (8.1–31.6)	**< 0.001**
PCT (ng/mL)	0.08 (0.05–0.14)	0.07 (0.05–0.11)	0.15 (0.11–0.22)	**< 0.001**
LBP (μg/mL)	6.49 (5.13–8.22)	6.22 (5.08–7.69)	7.20 (5.31–10.00)	0.102
LPS (EU/mL)	0.93 (0.69–1.64)	0.89 (0.67–1.46)	1.56 (1.42–2.76)	**0.008**
LTA (ng/mL)	45 (37–221)	41 (35–190)	208 (187–299)	**0.008**
Detectable bacterial DNA	25 (43.1%)	14 (31.8%)	11 (78.6%)	**0.004**
ELF test	11.3 (10.4–12.3)	10.9 (10.2–11.7)	12.6 (11.7–13.3)	**< 0.001**
PIIINP (ng/mL)	18.0 (11.9–27.1)	15.0 (10.8–22.0)	31.0 (19.6–44.0)	**< 0.001**
HA (ng/mL)	216 (89–370)	189 (83–270)	375 (259–871)	**< 0.001**
TIMP‐1 (ng/mL)	301 (241–440)	267 (223–342)	556 (377–749)	**< 0.001**
LSM (kPa)	34.6 (21.3–62.2)	28.6 (16.8–40.6)	66.9 (51.7–75.0)	**< 0.001**
Platelet count (G/L)	92 (69–125)	96 (72–123)	89 (68–125)	0.728
vWF antigen (%)	255 (185–351)	226 (170–300)	341 (256–393)	**< 0.001**
P‐selectin expression (MFI)				
PAR‐1 stimulation (SFLLRN)	2080 (1540–2590)	2160 (1730–2780)	1540 (1240–2240)	**0.003**
PAR‐4 stimulation (AYPGKF)	708 (167–1830)	857 (312–2410)	370 (88–841)	**0.003**
Epinephrine stimulation	12.1 (0.0–29.6)	13.4 (0.0–36.2)	7.6 (0.0–26.5)	0.333
Activated GPIIb/IIIa (MFI)				
PAR‐1 stimulation (SFLLRN)	8.3 (0.0–28.1)	14.6 (2.4–29.5)	0.0 (0.0–11.8)	**0.006**
PAR‐4 stimulation (AYPGKF)	6.5 (0.0–36.8)	9.9 (0.0–40.2)	0.0 (0.0–7.8)	**0.001**
Epinephrine stimulation	6.9 (0.0–24.0)	7.4 (0.0–24.2)	0.7 (0.0–17.4)	0.244

*Note:* Data presented as number n (% of available data) or median (IQR). *p*‐values in bold indicate statistical significance. Missing data: AST/ALT/CRP missing in *n* = 1; LPS/LTA/detectable bacterial DNA missing in *n* = 48; PIIINP/HA/TIMP‐1 missing in *n* = 2; LSM missing or invalid (IQR/median > 30%) in *n* = 22; vWF antigen missing in *n* = 2; P‐selectin and activated GPIIb/IIIa to epinephrine missing in *n* = 3; P‐selectin and activated GPIIb/IIIa to PAR‐4 missing in *n* = 1.

Abbreviations: ALD, alcohol‐related liver disease; ALT, alanine transaminase; AST, aspartate transaminase; BMI, body mass index; CRP, C‐reactive protein; ELF test, enhanced liver fibrosis score; GDF‐15, growth differentiation factor 15; GPIIb/IIIa, glycoprotein IIb/IIIa; HA, hyaluronic acid; HVPG, hepatic venous pressure gradient; IL‐6, interleukin 6; INR, international normalised ratio; LBP, lipopolysaccharide‐binding protein; LPS, lipopolysaccharide; LSM, liver stiffness measurement by vibration‐controlled transient elastography; LTA, lipoteichoic acid; MASLD, metabolic dysfunction‐associated steatotic liver disease; MELD, model for end‐stage liver disease; MFI, median fluorescence intensity; PAR, protease‐activated receptor; PCT, procalcitonin; PIIINP, procollagen III amino‐terminal peptide; TIMP‐1, tissue inhibitor of metalloproteinase‐1; vWF, von Willebrand factor.

### 3.2 Correlation Between GDF‐15 and Liver Disease‐Driving Mechanisms

**FIGURE 1:**
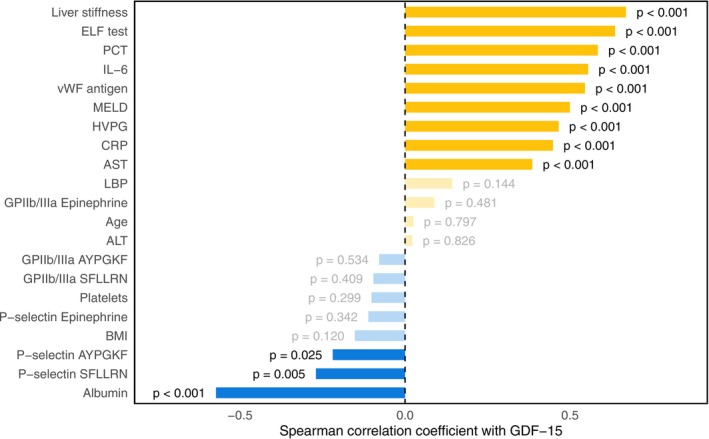
Correlation analysis between GDF‐15 and markers of hepatic dysfunction, fibrosis, portal hypertension, systemic inflammation, bacterial translocation, and platelet function. Analysis conducted using Spearman's correlation coefficients.

Higher GDF‐15 levels correlated with more pronounced liver disease severity, portal hypertension, systemic inflammation, liver stiffness, and fibrogenesis (Figure 1).

**FIGURE 2:**
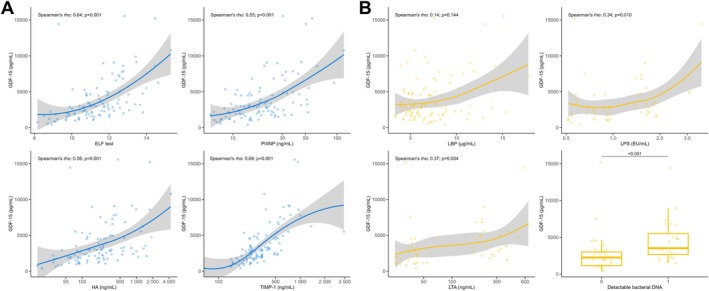
Association between GDF‐15 and biomarkers of (A) fibrogenesis and (B) bacterial translocation. Correlation analyses performed by Spearman's rank correlation and group comparison conducted by Mann–Whitney *U* test.

Specifically, GDF‐15 correlated positively with MELD (Spearman's *ρ*: 0.50), HVPG (*ρ*: 0.47), C‐reactive protein (CRP; *ρ*: 0.45), interleukin‐6 (IL‐6; *ρ*: 0.55), procalcitonin (PCT; *ρ*: 0.58), and LSM (*ρ*: 0.67), and negatively with albumin (*ρ*: −0.57) (all *p* < 0.001). Furthermore, GDF‐15 was linked to a higher enhanced liver fibrosis (ELF) test (*ρ*: 0.64; *p* < 0.001) and higher levels of its individual components (Figure 2A). Patients with prior decompensation had significantly higher levels than compensated patients (3330 [2310–5710] vs. 2150 [1360–3010] pg/mL; *p* < 0.001).

In a multivariable linear regression model including HVPG, MELD, albumin, IL‐6 (log_10_‐transformed) and ELF test (Table S1, Model 1), only ELF remained independently associated with (log10‐transformed) GDF‐15 (coefficient: 0.078; *p* = 0.005). After back‐transformation of the log‐scale, this translates to a 20% increase in GDF‐15 per 1‐point increase in ELF.

While substituting CRP for IL‐6 in the multivariable model revealed no significant association (*p* = 0.263; Table S1, Model 2), replacing IL‐6 with PCT showed a significant link between PCT and GDF‐15 (*p* = 0.009; Table S1, Model 3). Furthermore, LBP was linked to significantly higher GDF‐15 in the multivariable model (*p* = 0.002; Table S1, Model 4). Associations between ELF and GDF‐15 remained significant across all models.

In patients with available advanced biomarkers of bacterial translocation (54.7% of cohort), GDF‐15 was linked to higher LPS (*ρ*: 0.34; *p* = 0.010) and LTA levels (*ρ*: 0.37; *p* = 0.004) and was significantly higher in patients with detectable bacterial DNA in blood (3520 [2660–5520] vs. 2250 [1170–3020] pg/mL; *p* < 0.001) (Figure 2B). LPS and LTA remained significantly linked to GDF‐15 in multivariable models including HVPG, MELD, albumin, and ELF. Of note, baseline characteristics did not differ between patients with/without available data on bacterial translocation, except for higher HVPG values in those with available data (Table S2).

Across aetiologies, median GDF‐15 levels were highest in patients with ALD (3420 pg/mL) and lowest in patients with viral hepatitis (1640 pg/mL; *p* < 0.001). However, after accounting for baseline differences (Table S3) in HVPG, MELD, and IL‐6 in a multivariable linear regression model, no significant differences across aetiologies were found. Of note, among the 52 patients with ALD, 43 were abstinent at baseline, whereas 9 reported ongoing alcohol intake [31]. GDF‐15 levels tended to be higher in patients with ongoing alcohol consumption compared with abstinent patients (6580 [2540–7860] vs. 3240 [2290–5520] pg/mL; *p* = 0.364).

GDF‐15 did not correlate with age (*ρ*: 0.03; *p* = 0.797) and levels did not differ between male and female patients (2960 [1870–4820] vs. 2420 [1750–4630] pg/mL; *p* = 0.410). Furthermore, there was only a numerically negative association between GDF‐15 levels and body mass index (BMI) in the overall cohort (*ρ*: −0.15; *p* = 0.120) and in the subgroup of compensated patients (*ρ*: −0.20; *p* = 0.201).

### 3.3 Correlation Between GDF‐15 and Platelet Activation

While GDF‐15 levels correlated inversely with P‐selectin expression after PAR‐1 (*ρ*: −0.27; *p* = 0.005; Figure 1) and PAR‐4 stimulation (*ρ*: −0.22; *p* = 0.025; Figure 1) within the univariable analysis, no association was found after accounting for HVPG, MELD, albumin, IL‐6 and ELF test (PAR‐1 *p* = 0.496; PAR‐4 *p* = 0.543). GDF‐15 levels were comparable in patients with detectable (*n* = 75) and undetectable platelet surface P‐selectin expression in response to epinephrine (2960 [1820–4820] vs. 2860 [1970–4950] pg/mL; *p* = 0.844), and there was no significant correlation with GDF‐15 in patients with detectable levels (*ρ*: −0.11; *p* = 0.342).

GDF‐15 levels did not correlate with platelet surface expression of activated GPIIb/IIIa after PAR‐1 (*ρ*: −0.10; *p* = 0.409), PAR‐4 (*ρ*: −0.08; *p* = 0.534) or epinephrine stimulation (*ρ*: 0.09; *p* = 0.481) in patients with detectable expression levels (Figure 1). However, GDF‐15 was higher in patients with undetectable activated GPIIb/IIIa expression in response to PAR‐1 (4690 [2800–7060] vs. 2420 [1660–3670] pg/mL; *p* < 0.001), PAR‐4 (4510 [2630–6910] vs. 2320 [1670–3490] pg/mL; *p* < 0.001) and epinephrine stimulation (3260 [2350–6760] vs. 2520 [1610–4490] pg/mL; *p* = 0.015).

### 3.4 Impact of GDF‐15 on the Risk of Hepatic Decompensation

Over a median follow‐up of 51.5 (IQR: 26.0–58.2) months, first/further hepatic decompensation occurred in 38 patients, with 13 events of first decompensation (*n* = 12/13 new‐onset ascites) and 25 events of further decompensation (*n* = 13/25 ascites‐related complications).

**FIGURE 3:**
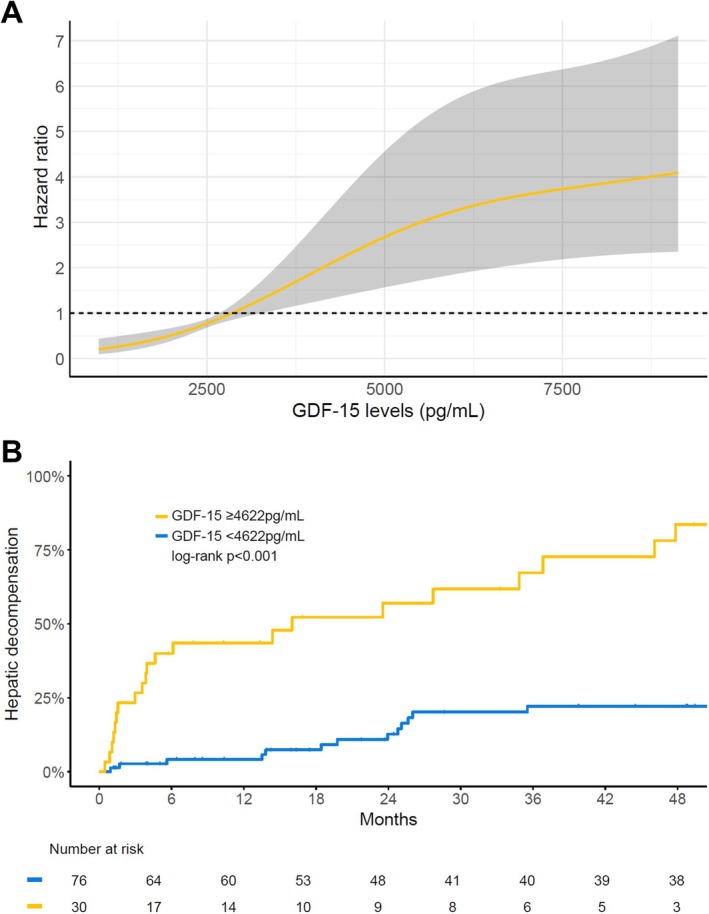
Impact of GDF‐15 on the risk of first/further hepatic decompensation. (A) Hazard ratio of GDF‐15 for first/further decompensation and (B) impact of high GDF‐15 levels (≥ 4622 pg/mL) on the risk of first/further decompensation. Hazard ratios modelled by restricted cubic splines and three degrees of freedom, and time‐to‐decompensation compared by log‐rank test.

Higher GDF‐15 was associated with a significantly higher risk of first/further decompensation in the univariable analysis (hazard ratio [HR] per 100 pg/mL: 1.020; 95% CI: 1.013–1.028; *p* < 0.001) (Figure 3A). This association remained significant in a multivariable model including HVPG, MELD, albumin and IL‐6 with an adjusted HR of 1.015 (per 100 pg/mL GDF‐15; 95% CI: 1.004–1.026; *p* = 0.007) (Table 2).

**TABLE 2:** Univariable and multivariable Cox regression models assessing the impact of GDF‐15 on the risk of (further) decompensation.

Covariables	Univariable analysis			Multivariable analysis		
	HR	95% CI	*p*	aHR	95% CI	*p*
HVPG (per mmHg)	1.108	1.047–1.172	**< 0.001**	1.067	0.998–1.141	0.057
MELD (per point)	1.123	1.049–1.202	**< 0.001**	0.950	0.848–1.063	0.370
Albumin (per g/L)	0.868	0.819–0.920	**< 0.001**	0.934	0.858–1.017	0.116
IL‐6 (per 10 pg/mL)	1.303	1.145–1.483	**< 0.001**	1.055	0.824–1.352	0.669
GDF‐15 (per 100 pg/mL)	1.020	1.013–1.028	**< 0.001**	1.015	1.004–1.026	**0.007**

*Note:* Univariable and multivariable Cox regression models assessing predictors of first or further hepatic decompensation. *p*‐values in bold indicate statistical significance.

Abbreviations: GDF‐15, growth differentiation factor 15; HVPG, hepatic venous pressure gradient; IL‐6, interleukin 6; MELD, model for end‐stage liver disease.

To identify patients at high risk of decompensation, GDF‐15 levels ≥ 4622 pg/mL yielded the highest discriminative value. Thirty patients (28.3%) had GDF‐15 values above this threshold (Table 1), which was associated with a significantly higher decompensation risk (HR: 7.132; 95% CI: 3.594–14.15; *p* < 0.001) (Figure 3B).

### 3.5 Impact of GDF‐15 on All‐Cause Mortality and Liver‐Related Death

**FIGURE 4:**
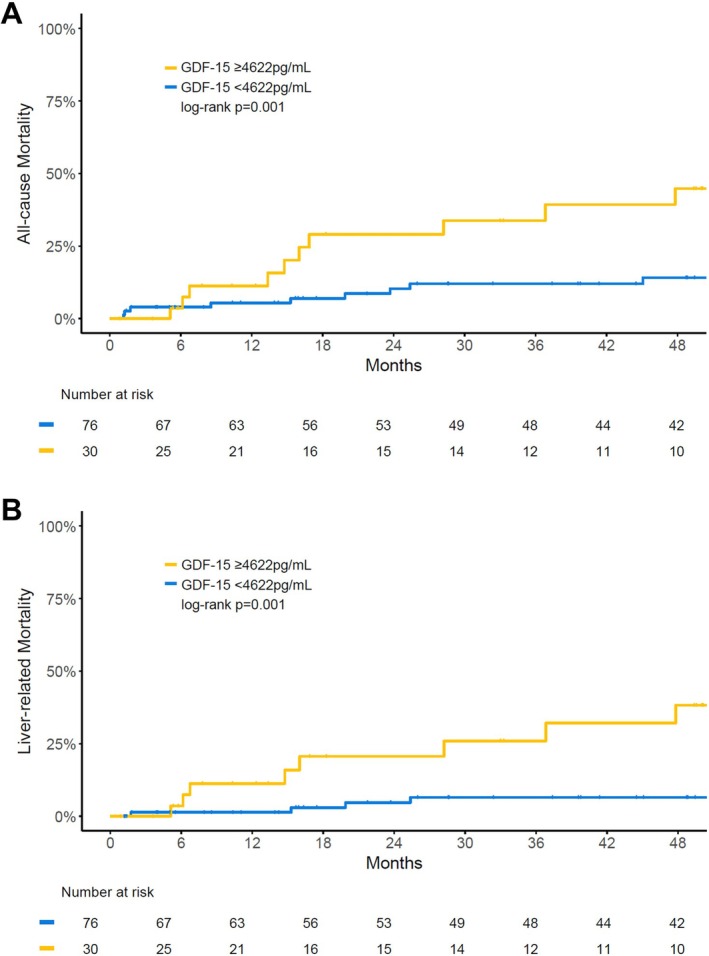
Impact of high GDF‐15 levels (≥ 4622 pg/mL) on the risk of (A) all‐cause mortality and (B) liver‐related mortality. Analyses conducted by log‐rank test.

During follow‐up, 21 patients died (19.8%; 61.9% liver‐related) and 10 patients underwent liver transplantation. In univariable analysis, higher GDF‐15 levels were associated with a significantly higher risk of all‐cause mortality (HR per 100 pg/mL: 1.019; 95% CI: 1.009–1.029; *p* < 0.001), with a particularly high risk in patients with GDF‐15 levels ≥ 4622 pg/mL (HR: 3.800; 95% CI: 1.598–9.036; *p* = 0.003) (Figure 4A). Higher GDF‐15 levels remained independently linked to the risk of all‐cause mortality in multivariable models adjusted for age and liver disease‐driving parameters. Specifically, in models including age as well as HVPG and albumin (Model 1), HVPG and IL‐6 (Model 2), or albumin and IL‐6 (Model 3), the adjusted HR per 100 pg/mL increase in GDF‐15 was 1.014 (*p* = 0.031), 1.014 (*p* = 0.021), and 1.013 (*p* = 0.040), respectively (Table 3).

**TABLE 3:** Univariable and multivariable Cox regression models assessing the impact of GDF‐15 on all‐cause mortality.

Covariables	Univariable analysis			Multivariable model 1		
	HR	95% CI	*p*	aHR	95% CI	*p*
Age (per year)	1.021	0.983–1.060	0.281	1.023	0.984–1.064	0.252
HVPG (per mmHg)	1.088	1.007–1.174	**0.032**	1.046	0.956–1.143	0.328
Albumin (per g/L)	0.879	0.815–0.947	**< 0.001**	0.939	0.858–1.028	0.175
IL‐6 (per 10 pg/mL)	1.316	1.113–1.557	**0.001**	—	—	—
GDF‐15 (per 100 pg/mL)	1.019	1.009–1.029	**< 0.001**	1.014	1.001–1.026	**0.031**

Covariables	Multivariable model 2			Multivariable model 3		
	aHR	95% CI	*p*	aHR	95% CI	*p*
Age (per year)	1.024	0.986–1.064	0.216	1.018	0.981–1.056	0.353
HVPG (per mmHg)	1.061	0.977–1.153	0.160	—	—	—
Albumin (per g/L)	—	—	—	0.933	0.839–1.037	0.198
IL‐6 (per 10 pg/mL)	1.132	0.924–1.387	0.231	1.045	0.803–1.360	0.745
GDF‐15 (per 100 pg/mL)	1.014	1.002–1.027	**0.021**	1.013	1.001–1.026	**0.040**

*Note:* Univariable and multivariable Cox regression models assessing predictors of all‐cause mortality. *p*‐values in bold indicate statistical significance.

Abbreviations: GDF‐15, growth differentiation factor‐15; HVPG, hepatic venous pressure gradient; IL‐6, interleukin 6.

With regard to liver‐related mortality, there was a significantly higher risk in patients with higher GDF‐15 levels (HR per 100 pg/mL: 1.019; 95% CI: 1.007–1.032; *p* = 0.002) with a pronouncedly higher risk in patients with GDF‐15 ≥ 4622 pg/mL (HR: 5.410; 95% CI: 1.756–16.668; *p* = 0.003) (Figure 4B). GDF‐15 remained an independent predictor of liver‐related death in multivariable models adjusted for HVPG, albumin, or IL‐6 (Table S4).

The association between GDF‐15 and HCC, extrahepatic malignancies, major adverse cardiovascular events, and bleeding events during follow‐up is discussed in the Supporting Informations.

## 4 Discussion

GDF‐15 is induced by cellular stress and inflammation and has been implicated in the pathogenesis and progression of various chronic diseases, including liver disease. In this study, we investigated the association between GDF‐15 and liver disease‐driving mechanisms in patients with cirrhosis. In our well‐characterised prospective cohort, we found that GDF‐15 is linked to fibrogenesis and bacterial translocation and predicts disease progression and mortality independently of hepatic dysfunction and portal hypertension.

From a pathophysiological perspective, GDF‐15 is induced by a wide variety of liver injury stimuli, ranging from toxins [21] to surgical partial‐hepatectomy [32] and metabolic dysfunction [22], and can even be directly induced by proinflammatory cytokines [33]. Accordingly, prior studies have demonstrated higher GDF‐15 levels in advanced fibrosis [16, 17] and in patients with ongoing hepatic necroinflammation [20, 34, 35]. Our results corroborate these findings, linking GDF‐15 to hepatic dysfunction, portal hypertension severity, liver stiffness, and fibrogenesis. Additionally, we observed positive associations with systemic inflammation and bacterial translocation, with LBP and PCT independently linked to elevated GDF‐15. In exploratory analyses, GDF‐15 was also higher in patients with detectable bacterial DNA in blood and correlated with both LPS and LTA as markers of gram‐negative and gram‐positive bacteria, respectively [36]. This aligns with previous data linking GDF‐15 to LPS and CRP levels in patients with alcohol‐associated hepatitis [20], as well as experimental data showing GDF‐15 upregulation following LPS injection in mice, mimicking bacterial infections [2].

However, whether GDF‐15 drives disease progression or represents a compensatory defence mechanism that is progressively upregulated remains unclear. Previous studies in mouse models of liver fibrosis showed that a neutralising antibody blocking GDF‐15 reduces hepatic stellate cell activation via TGF‐β‐signalling [37], a key profibrogenic pathway in liver disease [38]. Similarly, incubating hepatic stellate cells with GDF‐15 in vitro increased expression of profibrogenic markers [16]. However, direct hepatic effects of GDF‐15 remain controversial, as its only currently known receptor, glial cell‐derived neurotrophic factor receptor alpha‐like, is limited to two regions in the brainstem [39]. Furthermore, available insights are clouded by the fact that some commercially available batches of recombinant GDF‐15 had been contaminated with TGF‐β [40].

Conversely, arguments for GDF‐15 as a surrogate biomarker showing a compensatory increase with disease severity yet without negative effects can be found in preclinical models of metabolic dysfunction‐associated steatohepatitis [22] and toxin‐induced liver disease [21, 41], where GDF‐15 knockout led to a worsening of hepatic steatosis, inflammation, and fibrosis, while recombinant GDF‐15 [21] treatment or GDF‐15 overexpression [22, 41] attenuated it. The proposed mechanisms of these effects that may indirectly ameliorate liver disease progression range from the modulation of systemic inflammation via metabolic reprogramming of macrophages [41], the promotion of an anti‐inflammatory type 2 immune response [42], tissue tolerance in proinflammatory states [2] and decreased proinflammatory cytokine production [41, 43] to reduced profibrotic gene expression [41] and increased thermogenesis and lipolysis [44].

Despite the observed preclinical effects of GDF‐15 in liver disease, insights into prognostic implications are scarce. In a recent study by Rubio‐Tomás et al. [20], GDF‐15 significantly correlated with disease severity in alcohol‐related hepatitis and predicted steroid response and 90‐day survival. Furthermore, Kumazaki et al. [19] linked higher GDF‐15 levels to an increased risk of liver cancer and hepatic decompensation in metabolic dysfunction‐associated steatotic liver disease. Our study now expands the evidence on the prognostic role of GDF‐15 to patients with cirrhosis by demonstrating a significant link between elevated GDF‐15 and the risk of hepatic decompensation—independently from hepatic dysfunction, portal hypertension severity, as assessed by gold‐standard HVPG measurements, and systemic inflammation. Importantly, GDF‐15 levels were also independently linked to the risk of all‐cause and liver‐related mortality.

As for implications of GDF‐15 on the risk of de novo HCC and extrahepatic malignancies, we did not observe a significant association in our cohort. This contrasts prior findings demonstrating the role of GDF‐15 in (hepato‐) carcinogenesis [5, 6, 45, 46] due to its hypothesised influence on multiple relevant pathomechanisms including cell proliferation [35] and immunotolerance [47]. However, the low number of malignancies during the observation period has to be considered and further studies are clearly required to elucidate carcinogenic effects of GDF‐15 in cirrhosis.

Beyond the association between GDF‐15 and liver disease severity, we did not observe a significant link to platelet activation. However, GDF‐15 levels were significantly higher in patients where GPIIb/IIIa activation was not detectable in response to agonist stimulation, in line with previous data indicating that GDF‐15 can directly inhibit activation of GPIIb/IIIa on platelets [14]. Nevertheless, as a number of factors can influence platelet function in cirrhosis [25, 48], our findings must be interpreted with caution. Furthermore, in contrast to findings in cardiovascular research [10, 11], we did not observe a significant association of GDF‐15 with bleeding events after accounting for portal hypertension severity, as most events were classified as portal hypertensive bleeding.

With respect to potential limitations, we did not assess genetic polymorphisms that have been shown to influence GDF‐15 levels and thus cannot exclude small variations [49]. Second, no major adverse cardiovascular events occurred in our cohort, which could be a consequence of the low prevalence of cardiovascular risk factors as opposed to the comparably pronounced liver disease severity in our patients. Thus, the impact of GDF‐15 on cardiovascular risk in the setting of cirrhosis remains to be fully elucidated. Third, due to the in‐depth characterisation of our cohort including HVPG and platelet function measurements, the number of included patients was limited, and larger studies are needed to validate our findings. Along the same lines, advanced biomarkers of bacterial translocation were only available in a subset of the cohort, yet baseline characteristics were comparable between patients with/without available data. Lastly, despite our analyses of potential pathophysiological implications of GDF‐15, further research is required to fully elucidate its cellular origin and molecular function in liver disease and fibrosis.

In conclusion, this study highlights GDF‐15 as a promising and easily available blood‐based biomarker in patients with cirrhosis which reflects key disease‐driving pathomechanisms including fibrogenesis and bacterial translocation. Prognostically, elevated GDF‐15 levels predicted hepatic decompensation and mortality independently of the severity of liver disease and portal hypertension and could therefore be used for personalised treatment decisions. Nevertheless, further mechanistic studies are required to fully elucidate its role in liver disease.

## Author Contributions

All authors contributed either to study concept and design (B.S.H., T.R., T.G.) and/or data acquisition (all authors), analysis (B.S.H., T.R., T.G.), or interpretation (all authors). B.S.H., T.R., and T.G. drafted the manuscript, which was critically revised by all other authors. All authors read and approved the final manuscript.

## Funding

B.S.H., B.S., P.S. and T.R. are co‐supported by the Austrian Federal Ministry for Digital and Economic Affairs, the National Foundation for Research, Technology and Development, Boehringer Ingelheim, and the Christian Doppler Research Association. The study was supported by the Clinical Research Group MOTION, Medical University of Vienna, Vienna, Austria—a project funded by the Clinical Research Groups Programme of the Ludwig Boltzmann Gesellschaft (Grant Nr: LBG_KFG_22_32) with funds from the Fonds Zukunft Österreich.

## Ethics Statement

The study was approved by the local ethics committee of the Medical University of Vienna (2317/2019) and conducted in accordance with the 1964 Declaration of Helsinki and its later amendments.

## Consent

All patients provided informed consent prior to study inclusion.

## Conflicts of Interest

B.S.H. received travel support from Ipsen and Falk. B.S. received travel support from AbbVie, Gilead and Falk. R.F. served as a speaker and/or consultant and/or advisory board member for AbbVie, Alfasigma, Johnson&Johnson, Lilly, MSD, Pfizer, Takeda and UCB, and received travel support and/or grants/research support from AbbVie and Johnson&Johnson. M.J. has received grant support from Gilead, served as speaker and consultant for Gilead, and has served as speaker for Echosens. P.S. received consulting fees from PharmaIN and travel support from Falk Pharma. M.M. served as a speaker and/or consultant and/or advisory board member for AbbVie, Echosens, Eli Lilly, Falk, Gilead, Ipsen, Takeda, and W.L. Gore & Associates and received travel support from AbbVie and Gilead as well as grants/research support from Echosens. T.R. received grant support from Abbvie, Boehringer Ingelheim, Gilead, Intercept/Advanz Pharma, MSD, Myr Pharmaceuticals, Philips Healthcare, Pliant, Siemens and W.L. Gore & Associates, speaking honoraria from Abbvie, Echosens, Gilead, Intercept/Advanz Pharma, Roche, MSD, W.L. Gore & Associates, consulting/advisory board fee from Abbvie, Astra Zeneca, Bayer, Boehringer Ingelheim, Gilead, Intercept/Advanz Pharma, MSD, Resolution Therapeutics, Siemens; and travel support from Abbvie, Boehringer Ingelheim, Dr. Falk Pharma, Gilead and Roche. T.G. received speaker fees from Amgen, Bayer, Boehringer‐Ingelheim, Bristol Myers Squibb, Daiichi‐Sankyo, Novartis, and Pfizer, and grant support from Boehringer‐Ingelheim, Bristol Myers Squibb, Medtronic, and Abbott. T.P., K.B., L.B., M.H. G.K., E.C., B.E., S.L., K.Z., B.B., B.R.‐P, P.T., C.S., L.H., and S.P. were declare no conflicts of interest.

## Supporting information


**Figure S1:** Patient selection process.
**Table S1:** Multivariable linear regression models assessing the association between GDF‐15 and liver disease‐driving parameters.
**Table S2:** Baseline characteristics stratified by the availability of advanced biomarkers of bacterial translocation.
**Table S3:** Differences at baseline across liver disease aetiologies.
**Table S4:** Univariable and multivariable Cox regression models assessing the impact of GDF‐15 on liver‐related mortality.

## Data Availability

Pseudonymised data are available with publication upon reasonable request to the corresponding authors after approval of the proposal by the co‐authors and the Data Clearing House of the Medical University of Vienna in accordance with local data protection regulations.
