# Supplementary material for: GDF‐15 Levels in Cirrhosis Are Linked to Hepatic Fibrogenesis, Bacterial Translocation, and Worse Clinical Outcomes
Source: Liver Int. 2026 Jan 19;46(2):e70516. doi: 10.1111/liv.70516 (PMC12816942; doi:10.1111/liv.70516)
Supplement: Supplementary file 1 — Figure S1: Patient selection process. Table S1: Multivariable linear regression models assessing the association between GDF‐15 and liver disease‐driving parameters. Table S2: Baseline characteristics stratified by the availability of advanced biomarkers of bacterial translocation. Table S3: Differences at baseline across liver disease aetiologies. Table S4: Univariable and multivariable Cox regression models assessing the impact of GDF‐15 on liver‐related mortality. [file LIV-46-0-s001.pdf]

SUPPLEMENTARY MATERIAL

Hofer BS et al.

GDF-15 levels in cirrhosis are linked to hepatic fibrogenesis, bacterial translocation, and worse clinical outcomes

Correspondence (shared):

Thomas GREMMEL, MD, MBA

Department of Internal Medicine I, Cardiology and Intensive Care Medicine

Landesklinikum Mistelbach-Gänserndorf

Liechtensteinstraße 67, 2130 Mistelbach

P: +43 2572 9004 12810 F: +43 2572 9004 49245

M: thomas.gremmel@mistelbach.lknoe.at

Thomas REIBERGER, MD

Division of Gastroenterology and Hepatology

Department of Medicine III, Medical University of Vienna

Währinger Gürtel 18-20, 1090 Vienna, Austria

P: +4314040065890 F: +4314040047350

M: thomas.reiberger@meduniwien.ac.at

Table of Contents

Supplementary Methods. Assessment of bacterial translocation. 3

Supplementary Methods. Assessment of platelet function by flow cytometry. 4

Supplementary Methods. Definitions of first and further decompensation. 5

Supplementary Methods. Statistical analysis. 6

Supplementary Data. Association of GDF-15 levels with the incidence of hepatocellular carcinoma, extrahepatic malignancies, major adverse cardiovascular events and bleeding events. 7

Supplementary Table 1. Multivariable linear regression models assessing the association between GDF-15 and liver disease-driving parameters. 8

Supplementary Table 2. Baseline characteristics stratified by the availability of advanced biomarkers of bacterial translocation. 9

Supplementary Table 3. Differences at baseline across liver disease aetiologies. 10

Supplementary Table 4. Univariable and multivariable Cox regression models assessing the impact of GDF-15 on liver-related mortality. 11

Supplementary Fig. 1. Patient selection process. 12

Supplementary References. 13

Supplementary Methods. Assessment of bacterial translocation.

Advanced biomarkers of bacterial translocation in blood included lipopolysaccharide (LPS), lipoteichoic acid (LTA) and the presence of bacterial DNA.

LPS concentrations were measured by quantitative chromogenic limulus amebocyte lysate assay (BioWhittaker, Nottingham, UK) with a detection limit of 0.25 UE/mL. LTA levels were determined with a human LTA ELISA kit (Abbexa Ltd., Cambridge, UK) according to a previously established protocol.[1] The presence of bacterial DNA was assessed by broad-range PCR targeting the 16S rRNA gene, as described previously[2], and defined by a concentration of at least 5 pg/mL.

Supplementary Methods. Assessment of platelet function by flow cytometry.

Citrate-anticoagulated whole blood was diluted with phosphate-buffered saline (PBS) to achieve a final volume of 20µL with a concentration of $20x10^3$ platelets per µL.

Subsequently, samples were incubated with the platelet-specific monoclonal antibody anti-CD42b (clone HIP1, allophycocyanin labelled; Becton Dickinson, USA) for 10 minutes. Samples were then exposed to suboptimal concentrations of either (i) the protease-activated receptor (PAR)-1 agonist SFLLRN (final concentration: 14.25 µM, Bachem, Switzerland), (ii) the PAR-4 agonist AYPGKF (final concentration: 714 µM, Roche Diagnostics GmbH, Germany) or (iii) epinephrine (final concentration: 10 µM, MöLab, Germany) for 10 minutes in a dark environment. Used concentrations were previously titrated in 10 healthy controls to achieve 60-70% of the maximally possible increase in median fluorescence intensity (MFI).

After agonist stimulation, samples were incubated with antibodies for P-selectin (anti-CD62p-phycoerythrin, clone CLB- Thromb6; Immuno-tech, Beckman Coulter, USA) and activated GPIIb/IIIa (monoclonal antibody PAC-1-fluorescein, Becton Dickinson, USA) for 15 minutes. Isotype-matched control antibodies (Becton Dickinson, USA) were used to assess non-specific binding. Incubation was halted after 15 minutes by the addition of 500 µL of PBS.

Subsequent flow cytometric analyses were conducted using a FACS Canto II flow cytometer (Becton Dickinson, USA). Platelets were identified by forward scatter versus side scatter with a total of 10,000 events acquired within this gate, as well as with a platelet-specific monoclonal antibody anti-CD42b versus side scatter. MFI values were used to conduct subsequent analyses.

Supplementary Methods. Definitions of first and further decompensation.

According to Baveno VII criteria[3], first hepatic decompensation was defined as the first occurrence of overt ascites, overt hepatic encephalopathy (HE) or bleeding from oesophageal varices. Further decompensation was defined as (i) a second portal hypertension-related event (ascites, HE, variceal bleeding), (ii) recurrent ascites (≥3 paracenteses within 6 months), (iii) the occurrence of ascites-related complications (hepatorenal syndrome, spontaneous bacterial peritonitis), (iv) recurrent HE (≥2 episodes within 6 months) or (v) recurrent variceal bleeding. A worsening of the disease (i.e., additional events of further decompensation) in patients with prior further decompensation was included within the analysis.

Supplementary Methods. Statistical analysis.

Categorical variables were reported as absolute numbers and percentage and comparisons were conducted using Pearson's Chi-squared test or Fisher's exact test, as applicable. Continuous variables were reported as median and interquartile range, assessed for normal distribution by the Shapiro-Wilk test, and compared via independent samples t-test or Mann-Whitney U test, as applicable. Multiple group comparisons were conducted by one-way ANOVA or Kruskal-Wallis test, as applicable.

Supplementary Data. Association of GDF-15 levels with the incidence of hepatocellular carcinoma, extrahepatic malignancies, major adverse cardiovascular events and bleeding events.

Hepatocellular carcinoma (HCC) was diagnosed in 5 patients (4.7%) and extrahepatic malignancies in 9 patients (8.5%). Extrahepatic malignancies were primarily gastrointestinal cancers (n=3) or cancers of the upper airways and lung (n=2). GDF-15 levels (per 100 pg/mL) were neither associated with the risk of HCC (HR: 0.985; 95%CI: 0.937-1.035; p=0.539) nor with the risk of extrahepatic malignancies (HR: 1.009; 95%CI: 0.987-1.031; p=0.416) in univariable analyses.

Major adverse cardiovascular events (considering a composite endpoint of nonfatal stroke, nonfatal acute coronary syndrome, or cardiovascular death) did not occur in any of the patients over the course of the study, precluding further analyses.

Major bleeding events were defined by (i) a bleeding event resulting in death, (ii) symptomatic bleeding in a critical area, including intraspinal, intraocular, intracranial, retroperitoneal, pericardial, intraarticular, or intramuscular with compartment syndrome, (iii) bleeding leading to a drop in haemoglobin levels by at least 2 g/dL or necessitating the transfusion of ≥2 units of whole blood within two days of the bleeding event. Over the course of the study, major bleeding was documented in 16 patients. The majority of bleeding events occurred in the gastrointestinal tract and were classified as primarily related to the severity of portal hypertension (e.g., bleeding from oesophageal varices). Accordingly, while GDF-15 was linked to an increased bleeding risk within the univariable analysis (HR per 100 pg/mL: 1.017; 95%CI: 1.003-1.031; p=0.021), the association disappeared after adjusting for HVPG (aHR per 100 pg/mL: 1.011; 95%CI: 0.995-1.028; p=0.188).

Supplementary Table 1. Multivariable linear regression models assessing the association between GDF-15 and liver disease-driving parameters.

	Model 1 Including IL-6			Model 2 Including CRP		
**Parameter**	**Coefficient beta**	**Standard Error**	**p-value**	**Coefficient beta**	**Standard Error**	**p-value**
Intercept	2.720	0.483	<0.001	2.855	0.477	<0.001
HVPG	0.005	0.005	0.294	0.004	0.005	0.431
MELD	0.003	0.008	0.729	0.006	0.008	0.465
Albumin	-0.009	0.007	0.198	-0.010	0.007	0.135
ELF test	0.078	0.027	**0.005**	0.079	0.027	**0.005**
IL-6 (log-10)	0.094	0.078	0.235	-	-	-
CRP (log-10)	-	-	-	0.061	0.054	0.263
	**Model 3 Including PCT**			**Model 4 Including LBP**		
**Parameter**	**Coefficient beta**	**Standard Error**	**p-value**	**Coefficient beta**	**Standard Error**	**p-value**
Intercept	3.269	0.492	<0.001	2.392	0.475	<0.001
HVPG	0.004	0.005	0.431	0.007	0.005	0.131
MELD	0.006	0.007	0.440	0.005	0.007	0.496
Albumin	-0.009	0.006	0.159	-0.011	0.006	0.077
ELF test	0.060	0.028	**0.032**	0.082	0.026	**0.002**
PCT (log-10)	0.254	0.095	**0.009**	-	-	-
LBP (log-10)	-	-	-	0.462	0.145	**0.002**

Model 1: Multiple R-squared 0.47, adjusted R-squared 0.44, overall model p-value <0.001. Model 2: Multiple R-squared 0.47, adjusted R-squared 0.44, overall model p-value <0.001. Model 3: Multiple R-squared 0.50, adjusted R-squared 0.47, overall model p-value <0.001. Model 4: Multiple R-squared 0.51, adjusted R-squared 0.49, overall model p-value <0.001.

Abbreviations: GDF-15, growth differentiation factor 15; HVPG, hepatic venous pressure gradient; MELD, model for end-stage liver disease; ELF test, enhanced liver fibrosis score; IL-6, interleukin 6; CRP, C-reactive protein; PCT, procalcitonin; LBP, lipopolysaccharide-binding protein.

Supplementary Table 2. Baseline characteristics stratified by the availability of advanced biomarkers of bacterial translocation.

	Not available (n = 48)	Available (n = 58)	p-value
GDF-15 (pg/mL)	2950 (1880-5390)	2830 (1800-4520)	0.376
Age (years)	54.3 (46.2-61.4)	56.9 (46.6-63.2)	0.869
Sex (male)	38 (79.2%)	37 (63.8%)	0.083
BMI (kg/m^2)	26.5 (22.2-29.5)	25.7 (23.0-30.4)	0.740
Aetiology of cirrhosis			0.808
ALD	24 (50.0%)	28 (48.3%)	
Viral hepatitis	12 (25.0%)	12 (20.7%)	
Cholestatic disease	3 (6.3%)	5 (8.6%)	
MASLD	4 (8.3%)	3 (5.2%)	
Other	5 (10.4%)	10 (17.2%)	
Decompensated disease	27 (56.3%)	37 (63.8%)	0.429
HVPG (mmHg)	16 (11-19)	19 (14-21)	**0.020**
MELD	11 (9-14)	11 (9-15)	0.777
Child-Pugh stage (A/B/C)	62.5%/25.0%/12.5%	53.4%/37.9%/8.6%	0.346
Albumin (g/L)	37.8 (32.4-40.5)	36.8 (34.2-40.2)	0.934
Bilirubin (mg/dL)	1.16 (0.79-1.77)	1.08 (0.71-2.24)	0.871
INR	1.4 (1.2-1.5)	1.3 (1.2-1.5)	0.880
Creatinine (mg/dL)	0.72 (0.62-0.94)	0.72 (0.59-0.87)	0.475
Sodium (mmol/L)	139 (137-140)	139 (137-141)	0.433
AST (U/L)	41 (31-57)	38 (27-56)	0.428
ALT (U/L)	33 (24-47)	28 (20-38)	0.106
CRP (mg/dL)	0.24 (0.11-0.66)	0.26 (0.09-0.61)	0.470
IL-6 (pg/mL)	6.3 (4.1-17.0)	8.0 (3.4-12.0)	0.699
PCT (ng/mL)	0.10 (0.06-0.15)	0.07 (0.05-0.13)	0.140
LBP (µg/mL)	6.58 (5.42-8.60)	6.18 (4.92-7.76)	0.151
ELF test	11.3 (10.6-12.6)	11.2 (10.4-12.1)	0.360
LSM (kPa)	30.7 (16.7-61.0)	35.0 (27.7-62.8)	0.271
Platelet count (G/L)	97 (69-138)	89 (66-110)	0.176
vWF (%)	256 (184-368)	253 (188-323)	0.424

Data presented as number n (% of available data) or median (IQR). P-values in bold indicate statistical significance.

Abbreviations: GDF-15, growth differentiation factor 15; BMI, body mass index; ALD, alcohol-related liver disease; MASLD, metabolic dysfunction-associated steatotic liver disease; HVPG, hepatic venous pressure gradient; MELD, model for end-stage liver disease; INR, international normalised ratio; AST, aspartate transaminase; ALT, alanine transaminase; CRP, C-reactive protein; IL-6, interleukin 6; PCT, procalcitonin; LBP, lipopolysaccharide-binding protein; ELF test, enhanced liver fibrosis score; LSM, liver stiffness measurement by vibration-controlled transient elastography; vWF, von Willebrand factor.

Supplementary Table 3. Differences at baseline across liver disease aetiologies.

	**ALD (n = 52)**	**Cholestatic (n = 8)**	**MASLD (n = 7)**	**Viral (n = 24)**	**Other (n = 15)**	**p-value**
GDF-15 (pg/mL)	3420 (2310-6640)	2610 (1770-5390)	2200 (2040-2470)	1640 (1190-2450)	3360 (2820-4820)	**<0.001**
Age (years)	54.4 (47.1-62.6)	47.5 (38.5-61.0)	65.7 (57.9-69.6)	56.4 (46.5-63.2)	49.4 (41.8-60.8)	0.120
Sex (male)	42 (80.8%)	1 (12.5%)	5 (71.4%)	17 (70.8%)	10 (66.7%)	**0.004**
BMI (kg/m^2)	26.0 (23.1-29.2)	21.7 (19.6-24.8)	31.9 (29.8-33.0)	25.5 (23.2-28.7)	26.2 (21.6-30.5)	**0.007**
Compensated	8 (15.4%)	6 (75.0%)	7 (100.0%)	18 (75.0%)	3 (20.0%)	**<0.001**
HVPG (mmHg)	20 (17-22)	18 (16-20)	11 (6-13)	11 (8-15)	19 (15-22)	**<0.001**
MELD	14 (10-17)	9 (8-14)	9 (8-10)	9 (8-10)	11 (10-14)	**<0.001**
Albumin (g/L)	35.8 (31.2-39.5)	35.6 (34.6-36.0)	40.4 (39.5-41.5)	40.5 (39.3-43.7)	35.1 (30.7-38.4)	**<0.001**
CRP (mg/dL)	0.41 (0.16-0.88)	0.37 (0.16-0.81)	0.38 (0.13-0.60)	0.08 (0.04-0.13)	0.32 (0.21-0.60)	**<0.001**
IL-6 (pg/mL)	10.6 (6.2-19.2)	7.1 (4.3-18.7)	4.5 (2.4-5.6)	3.4 (1.5-4.7)	7.6 (4.6-15.9)	**<0.001**
PCT (ng/mL)	0.11 (0.06-0.15)	0.11 (0.07-0.22)	0.08 (0.06-0.10)	0.04 (0.03-0.06)	0.12 (0.08-0.20)	**<0.001**
LBP (µg/mL)	6.66 (5.30-8.09)	5.28 (4.45-6.72)	8.85 (6.67-11.00)	6.31 (5.22-7.32)	5.26 (4.40-7.99)	0.094
ELF test	11.9 (11.0-12.9)	11.4 (11.0-12.5)	10.0 (9.4-10.9)	10.4 (9.6-10.9)	12.0 (10.9-12.4)	**<0.001**

Data presented as number n (% of available data) or median (IQR). P-values in bold indicate statistical significance.

Abbreviations: ALD, alcohol-related liver disease; MASLD, metabolic dysfunction-associated steatotic liver disease; GDF-15, growth differentiation factor 15; BMI, body mass index; HVPG, hepatic venous pressure gradient; MELD, model for end-stage liver disease; CRP, C-reactive protein; IL-6, interleukin 6; LBP, lipopolysaccharide-binding protein; ELF test, enhanced liver fibrosis score.

Supplementary Table 4. Univariable and multivariable Cox regression models assessing the impact of GDF-15 on liver-related mortality.

	Univariable analysis			Multivariable model 1		
**Covariables**	**HR**	**95% CI**	**p-value**	**aHR**	**95% CI**	**p-value**
HVPG (per mmHg)	1.101	0.998-1.215	0.055	1.074	0.970-1.190	0.170
Albumin (per g/L)	0.928	0.838-1.028	0.151	-	-	-
IL-6 (per 10 pg/mL)	1.209	0.938-1.558	0.143	-	-	-
GDF-15 (per 100 pg/mL)	1.019	1.007-1.032	**0.002**	1.018	1.004-1.031	**0.011**
	**Multivariable model 2**			**Multivariable model 3**		
**Covariables**	**HR**	**95% CI**	**p-value**	**aHR**	**95% CI**	**p-value**
HVPG (per mmHg)	-	-	-	-	-	-
Albumin (per g/L)	0.984	0.884-1.096	0.771	-	-	-
IL-6 (per 10 pg/mL)	-	-	-	1.002	0.974-1.032	0.879
GDF-15 (per 100 pg/mL)	1.019	1.004-1.033	**0.010**	1.019	1.005-1.033	**0.007**

Univariable and multivariable Cox regression models assessing predictors of liver-related mortality. P-values in bold indicate statistical significance.

Abbreviations: HVPG, hepatic venous pressure gradient; IL-6, interleukin 6; GDF-15, growth differentiation factor 15.

Supplementary Fig. 1. Patient selection process.

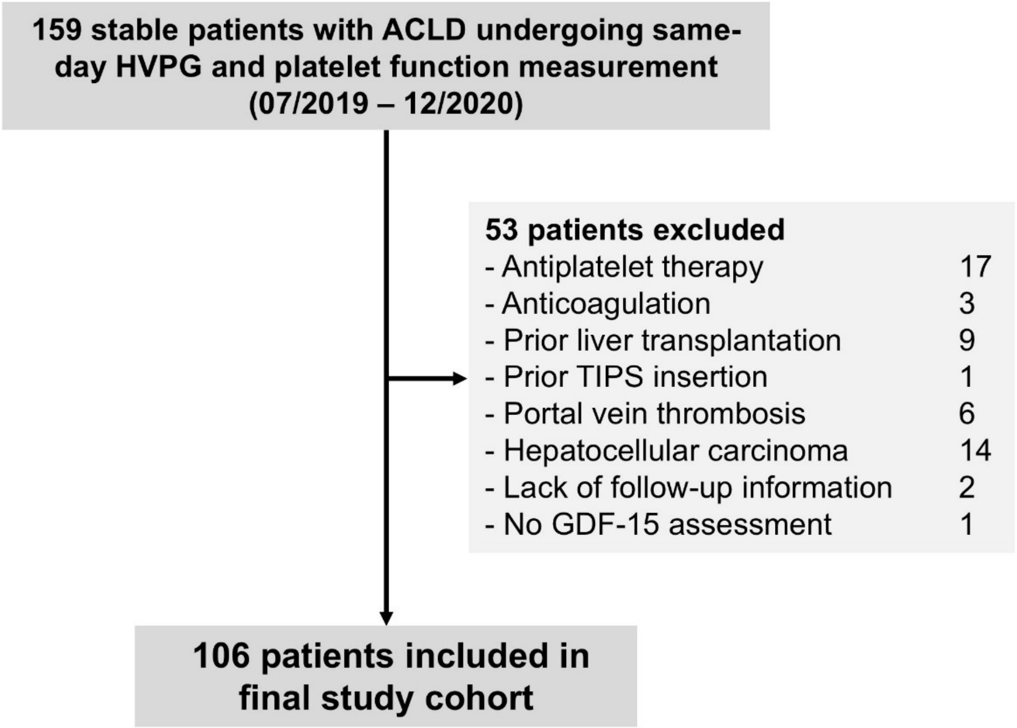

Abbreviations: ACLD, advanced chronic liver disease; HVPG, hepatic venous pressure gradient; TIPS, transjugular intrahepatic portosystemic shunt; GDF-15, growth differentiation factor 15.

Supplementary References.

1. Simbrunner B, Caparrós E, Neuwirth T, et al. Bacterial translocation occurs early in cirrhosis and triggers a selective inflammatory response. Hepatol Int. 2023 Aug;17(4):1045–56.

2. Francés R, Benlloch S, Zapater P, et al. A sequential study of serum bacterial DNA in patients with advanced cirrhosis and ascites. Hepatology. 2004 Feb;39(2):484–91.

3. de Franchis R, Bosch J, Garcia-Tsao G, et al. Baveno VII - Renewing consensus in portal hypertension. J Hepatol. 2022 Apr;76(4):959–74.
